# Hepatocellular Carcinoma: Risk Factors, Diagnosis and Treatment

**DOI:** 10.3889/oamjms.2015.111

**Published:** 2015-10-29

**Authors:** Dafina Janevska, Viktorija Chaloska-Ivanova, Vlado Janevski

**Affiliations:** 1 *University Clinic of Gastroenterohepatology, Faculty of Medicine, Ss Cyril and Methodius University of Skopje, Skopje, Republic of Macedonia*; 2 *University Clinic of Abdominal Surgery, Faculty of Medicine, Ss Cyril and Methodius University of Skopje, Skopje, Republic of Macedonia*

**Keywords:** hepatocellular carcinoma, risk factors, diagnosis, management, review article

## Abstract

Hepatocellular carcinoma (HCC) is the most often primary cancer of the liver and is one if the leading cause of cancer-related death worldwide. The incidence of HCC has geographic distribution with the highest levels in countries with developing economies. Patients with hepatocellular carcinoma have poor prognosis despite the achievements in surgery techniques and other therapeutic procedures and it is a reason why continuous attention should be paid to this issue.

This article provides an overview of this disease based on an extensive review of relevant literature. The article summarizes the current risk factors, diagnosis, staging and the management of HCC.

## Introduction

Liver cancer is the fifth most common cancer in men and the seventh in women [1]. World-wide incidence is between 250,000 and 1,000,000 new cases per year with a male-female ratio of about 4 or 5:1 [2]. Hepatocellular carcinoma (HCC) is the most often primary cancer of the liver, accounting from 85% till 90% of all primary liver cancers [3]. Its occurrence reaches a peak at approximately 70 years of age with a rarely occurrence before the age of 40 years. Major risk factors for HCC include chronic alcohol consumption, hepatitis B, hepatitis C and non-alcoholic fatty liver disease [3]. Other, less common causes are Wilson’s disease, hereditary hemochromatosis, alpha1-antitrypsin deficiency, primary biliary cirrhosis and autoimmune hepatitis [4, 5]. The incidence of HCC in different regions of the world varies due to the incidence of the risk factors. Such countries are China, sub-Saharan Africa, Hong Kong and Taiwan with more than 15 cases per 100,000 populations per year [6]. Any agent leading to chronic hepatic injury and eventually cirrhosis has been associated with HCC. Although multiple etiologic factors are responsible for HCC, it has been made a significant progress in these past years in the understanding of the molecular mechanisms and the pathogenesis of this disease. This article provides an overview of this disease based on our extensive review of relevant literature. We will summarize the current risk factors, diagnosis, staging and the management of HCC.

Global variation of the incidence of HCC is in relation with the complex etiology of the neoplasm and the literature data depends on the evaluated region and population, time in which the studies are done or the used methodologies.

**Figure 1:**
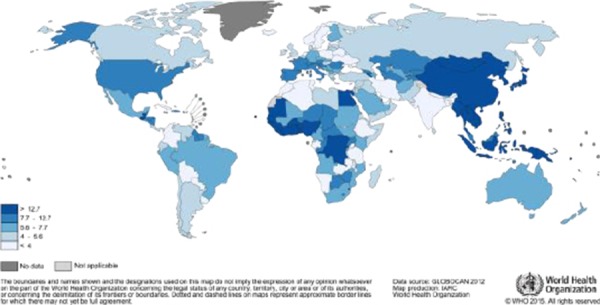
*Estimated Liver Cancer Incidence Worldwide in 2012: Men* (http://globocan.iarc.fr/old/FactSheets/cancers/liver-new.asp)

**Figure 2:**
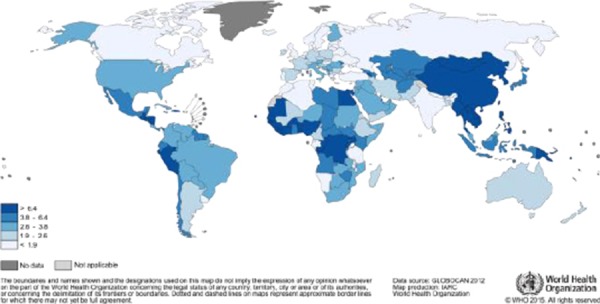
*Estimated Liver Cancer Incidence Worldwide in 2012: Women* (http://globocan.iarc.fr/old/FactSheets/cancers/liver-new.asp)

Relevant information can be provided by GLOBALCAN database maintained by the International Agency for Research on Cancer (Figure 1, 2) [7, 8].

## Risk factors

Almost 50% of all cases of HCC are associated with HBV infection and 25% are associated with HCV [9]. HBV is double-stranded DNA-containing virus that is able to integrate its DNA into the hepatic cells, act as a mutagenic agent and cause secondary chromosomal rearrangement and increasing genomic instability [10]. This is the reason why the risk of HCC development is 100-fold higher for patients who are infected with HBV in comparison with those who are not infected [11].

Major cause of HCC in Europe, Japan, Latin America and the United States is cirrhosis caused by hepatitis C virus infection and the annual incidence ranges from 2% to 8% [9, 10]. Generally HCV-infected patients have 17 fold higher risk of developing HCC than uninfected [11-13]. HCV is an RNA-containing virus, unable to integrate into the host genome and therefore causes HCC by various indirect mechanisms such as alterations in apoptotic pathways and tumor formation [5, 14].

Obesity and diabetes mellitus have been correlated with increased risk of HCC [15, 16]. These patients have twice higher chance of developing HCC compared to those who are not obese and do not have diabetes [17]. Alcohol is another important risk factor for developing HCC with increasing as levels of alcohol intake rise [16]. Chronic consumption of alcohol from 40 to 60 grams of alcohol on a daily bases are highly associated with HCC [18]. Aflatoxin is a mycotoxin produced by Aspergillus flavus and related fungus that contaminates stored foods such as rice, corn, soybeans, and peanuts. In some regions of the world, especially in Asia and Africa, it is major risk factors of HCC [19]. Chronic exposure to aflatoxin is highly associated with HCC due to damaging the DNA of hepatic cells and causing mutation of the p53 tumor suppressor gene [20]. Mutations of other genes and chromosome aberrations are described in HCC also. Point mutation of the c-KRAS gene and co-amplification of the cyclin D1 gene are rare and are detected in 3% and 11% of HCCs respectively. Mutations of beta-catenin gene are detected in 26-41% of HCCs. Frequent allelic losses are reported at loci 1p, 4q, 5q, 8p, 11p, 13q, 16p, 16q and 17p and loss of heterozygosity (LOH) is reported on chromosome 16. Reduction of expression of p21WAF/CIP1 (universal CDK inhibitor), P16 protein loss, high level expression of transforming growth factor-beta (TGF-beta), high expression of DNA metiltransferase (DNMT1) mRNA are found in significant percentage of HCCs.

There is increased risk of developing HCC in several rare inherited disorders: glycogen storage disease, alpha-1-antitrypsin deficiency, metal storage disease and chronic cholestatic syndromes [21].

## Diagnosis

In many patients, HCC is asymptomatic and when symptoms occur they are usually related to those of chronic liver disease such as yellowing of the skin and eyes, pain in the right upper abdominal side, swelling of the abdomen, weakness, weight loss, fever [19, 22]. Over the past decades more asymptomatic patient are being diagnosed as a result of the active surveillance and the increased awareness of HCC in high risk patients, especially in those with cirrhosis. Screening for HCC in these patients is recommended every six months [1] and most used tools are serum levels of alpha-fetoprotein (AFP) and ultrasound [22]. Also the role of the serum level of AFP not always correlate with the tumor growth and it has been showed that is less useful than previously tough [23]. Generally values over 400 ng/mL can confirm the diagnosis in 20% of HCC patients [19]. The diagnosis of HCC can be made by history, physical examination and using noninvasive imaging methods such as ultrasound, MRI and CT scan. Suspicious lesion in the liver detected by ultrasound usually requires additional imaging methods to confirm the diagnosis and to detect additional, smaller, lesions that previously were not seen. Specificity and sensitivity of the ultrasound depends on the size of the tumor, ability to detect tumors 3-5cm in diameter is 80-95% and 60-80% of tumors <1cm in diameter [5, 24]. Standard noninvasive diagnostic methods for diagnosing HCC are dynamic multiphasic multidetector-row CT (MDCT) and magnetic resonance imaging (MRI) [25]. Typical finding for HCC in contrast enhanced CT is arterial phase enhancement followed by loss of enhancement (washout) in the portal venous and delayed phases [26]. This image finding has a sensitivity of 90% and specificity of 95% for diagnosing HCC [26, 27]. Nodules less that 1 cm are difficult to assess and usually image-guided biopsy should be considered for these lesions [28]. This also should be done for focal hepatic masses with atypical imaging features or discrepant findings on CT and MRI, or for lesions detected in the absence of cirrhosis [1]. A negative result from the biopsy does not rule out malignancy and continuous surveillance and ultrasound check up is needed in intervals from 3 till 6 months until they grow in size or change their echo pattern [29].

## Treatment

**Figure 3:**
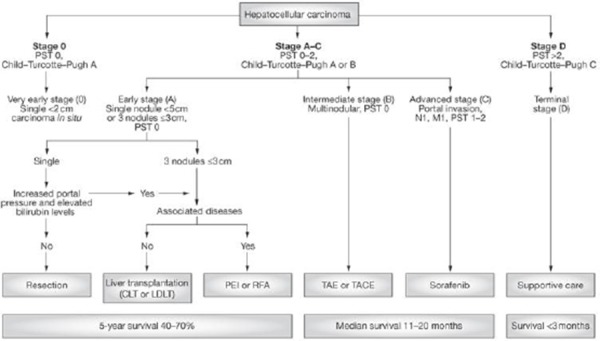
Barcelona Clinic Liver Cancer (BCLC) classification. *PST – Performance Status Test.

Tumor staging has an essential role in guiding and making decision for further treatment. There are many systems to use in patients with HCC, but today most effective and most used is the Barcelona Clinic Liver Cancer (BCLC) classification [30] (Figure 3).

**Figure 4:**
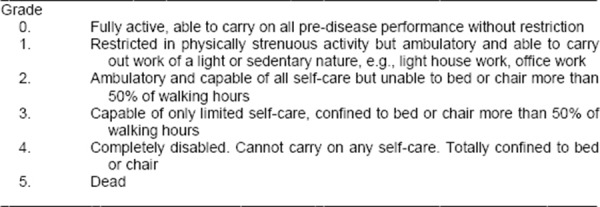
*Performance status*.

Performance status scale is developed by the Eastern Cooperative Oncology Group (ECOG) is included in BCLC system and is used to evaluate the progression of diseases (Figure 4).

**Figure 5:**
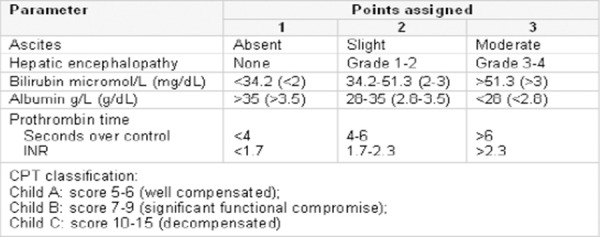
*Child-Turcotte-Pugh score*.

Child-Turcotte-Pugh score which is also included in BCLC is used to assess the prognosis of chronic liver disease (Figure 5).

Using the BCLC system can help the doctor classify the patients with early-stage HCC, who may benefit from curative therapies and the patients with advanced-stage disease who would need palliative treatment. Sadly majority of patients present with advanced HCC at the time of diagnosis and are not candidate for curative therapies. Depending of the stage of the disease there are several therapeutic choices: surgical resection, percutaneous ethanol injection (PEI), radiofrequency ablation (RFA), transarterial chemoembolization (TACE) and radio-embolization [31].

First option for patients who have optimal profile according to BCLC staging system should be resection [32]. Surgical resection is the choice of treatment for patients with single nodules, no underlying cirrhosis and good liver function [33]. Treatment with PEI and RFA are optimal treatment option for patients with small tumours who are not candidates for surgical resection or liver transplantation [26]. Treatment with radiofrequency ablation demonstrated that complete ablation of lesions smaller than 2 cm is possible in more than 90% of cases, with a local recurrence rate of less than 1% [32, 34]. Survival rates after this treatment is 100% at first year and 98% at second year [1]. Standard treatment for the patients with intermediate-stage HCC is TACE. Patients with compensated liver function (Child B up to 8 points), with large single nodule (< 5 cm) or multifocal HCC without evidence of vascular invasion or extra hepatic spread are considered candidates for TACE [33]. Also TACE plays an important role in the palliative treatment and it is a standard choice of treatment in patients with asymptomatic multinodular disease [35]. The prognosis of patients with HCC is poor in symptomatic patients in whom five-year survival rate is reported to be less than 5%. It is very poor in patients with AFP levels greater than 100ng/ml at the time of diagnosis, portal vein thrombosis and presence of p53 mutation. Long –term survival is reported only in patients with small, asymptomatic HCC (Table 1).

**Table 1:** Estimated Incidence, Mortality and Prevalence of liver cancer Worldwide in 2012. Estimated numbers (thousands) http://globocan.iarc.fr/old/FactSheets/cancers/liver-new.asp

	Men			Women			Both sexes		
	Cases	Deaths	5-year prev.	Cases	Deaths	5-year prev.	Cases	Deaths	5-year prev.
World	554	521	453	228	224	180	782	746	633

More developed regions	92	80	112	42	43	51	134	123	164

Less developed regions	462	441	341	186	182	129	648	622	469

WHO Africa region (AFRO)	25	24	17	14	13	9	39	37	26

WHO Americas region (PAHO)	40	35	35	23	23	18	63	58	53

WHO East Mediterranean region (EMRO)	20	19	12	10	9	6	29	28	18

WHO Europe region (EURO)	47	44	42	23	25	20	71	69	61

WHO South-East Asia region (SEARO)	55	52	33	25	24	15	80	77	48

WHO Western Pacific region (WPRO)	368	347	314	133	129	112	501	477	426

IARC membership (24 countries)	120	104	135	56	55	60	176	159	195

United States of America	23	17	21	8	7	7	30	24	27

China	293	282	220	101	101	71	395	383	291

India	17	17	8	10	10	5	27	27	13

European Union (EU-28)	36	32	33	16	17	14	52	48	47

The development of new drugs and types of therapy can improve the outcome of patients with hepatocellular carcinoma, but the key factor in management of HCC is still making a diagnosis in the very-early stages of disease and continuously surveillance of the patients with a high risk of HCC [1, 36, 37].
